# The Impact of an Implementation Project on Primary Care Staff Perceptions of Delivering Brief Alcohol Advice

**DOI:** 10.1155/2016/4731571

**Published:** 2016-06-30

**Authors:** Hanna Reinholdz, Preben Bendtsen, Fredrik Spak, Ulrika Müssener

**Affiliations:** ^1^Unit of Social Medicine, University of Gothenburg, 405 30 Gothenburg, Sweden; ^2^Department of Medical Specialist and Department of Medical and Health Sciences, Linköping University, 581 83 Motala, Sweden; ^3^Department of Medical and Health Sciences, Linköping University, 581 83 Linköping, Sweden

## Abstract

*Objective*. To explore how the perceptions and experiences of working with risky drinkers change over time among primary health care staff during a systematic implementation project.* Methods*. Qualitative focus group interviews took place before and after the implementation of the project.* Results*. The staff displayed a positive change during the implementation period with regard to awareness, knowledge, and confidence that led to a change in routine practice. Throughout the project, staff were committed to engaging with risky drinkers and appeared to have been learning-by-doing.* Conclusions*. The results indicated a positive attitude to alcohol prevention work but staff lack knowledge and confidence in the area. The more practical experience during the study is, the more confidence seems to have been gained. This adds new knowledge to the science of implementation studies concerning alcohol prevention measures, which have otherwise shown disappointing results, emphasizing the importance of learning in practice.

## 1. Introduction

Although the efficacy and effectiveness, as well as the cost-effectiveness, of screening and brief alcohol interventions (SBI) have been shown to be strong, the implementation of SBI into routine care practice has not been satisfactory; it seems to be difficult to motivate health care professionals to deliver SBI [1–6]. Many implementation projects have tried to overcome known barriers such as lack of time, resources, training, and negative attitudes to working with SBI with limited success [4, 5, 7–9]. In a survey in the United Kingdom comparing role security and therapeutic commitment between 1999 and 2009 among general practitioners (GPs), it was seen that the main issue was lack of therapeutic commitment [6]. The study also highlighted that lack of time and resources rather than negative attitudes was related to the lack of therapeutic commitment to SBI. Implementation of new methods has repeatedly been shown to need more than simple training sessions to be effective [4, 10]. Implementation research suggests that a multifaceted strategy addressing several barriers may be more effective than simple training sessions. In addition, implementation efforts involving more professionals than GPs can improve how professionals work together towards increased SBI activities [4, 10, 11].

In a recent study from five European countries involving 120 primary care units, no evidence that SBI rates were influenced by role security or therapeutic commitment was found [12]. Other factors, not specifically studied, such as clinical priorities and management support might be more important for implementation. This study underlines a review by Nilsen et al. [13], which concluded that motivation to engage in SBI should be viewed as a dynamic process encompassing the characteristics of the individual health professional, the patients, the clinical setting, and the wider context.

Thus, there is a knowledge gap on how to engage primary care staff in brief alcohol advice. How do we overcome the perceived lack of knowledge and reluctance to ask patients about their alcohol habits? Tentative answers might be found by giving office-based support material and management support rather than trying to change already positive attitudes among staff [4, 10]. How staff's performance changes over time if these preconditions are offered has not been sufficiently studied. Most previous researches have measured role security and therapeutic commitment at one point in time or just after a training session using standardized questionnaires [5, 10, 12]. The few studies that have measured role security and therapeutic commitment before and after an educational session do not provide information about how a change in engagement is accomplished or mediated [8, 14]. Thus, there is a lack of qualitative studies following staff practices over time in implementation projects aiming to establish new routines.

*Aim*. The aim of this study was to explore how the perceptions and experiences of working with risky drinkers change over time among primary health care staff during a systematic implementation project.

## 2. Methods

The study was part of the Swedish implementation study SPIRA (Secondary Prevention Implementation Research on Alcohol) that aimed to study the implementation process of different SBI methods at primary health care centres (PHCs) in Sweden. This study was approved by the Regional Ethical Review Board in Gothenburg, Sweden (405-10/2010).

The SPIRA study was conducted during 2010–2012 in 16 PHCs from three different regions in Sweden. In brief, the SPIRA study started with a baseline measurement of SBI rates over 2 days. After the baseline period, a 3-hour training session on how to perform SBI was given to all staff. It was then followed by the first implementation period of 4 weeks during which staff were asked to routinely offer SBI to their patients. Six months after the first implementation period, a booster education session of 1-2 hours was given followed by a second 4-week SBI implementation period. A significant increase in SBI rates was seen during the implementation periods, results reported elsewhere [15].

### 2.1. Materials and Participants

As part of the study, qualitative data were collected during a series of focus group interviews [16] with staff at baseline and 6 months after the second implementation period. For some participating units, the follow-up interviews were some months later. A total of 30 focus group interviews were included in this study; 16 were conducted at baseline and 14 at follow-up. Two of the PHCs did not participate in the follow-up because they were recruited too late, which explains why there are fewer interviews for the follow-up.

The PHCs included in the SPIRA study were located in three counties, selected to ensure representation from various parts of Sweden and to include both rural and urban areas. All PHCs in these counties were invited to participate in the study. Because we did not reach the intended number of PHCs with this procedure, we proceeded with snowball sampling using our research networks. We aimed to explore how the perceptions and experiences of working with risky drinkers change over time among primary health care staff during a systematic implementation project; therefore some of the staff were allowed to refrain from participating in the implementation.

All staff who had actively participated in the implementation process were invited to participate in the focus group interviews. The individuals who volunteered to participate were then given more information about the aim and content of the study. Reasons for not participating included sick leave, vacation, terminated employment, lack of time, or other commitments at the PHC. Our aim was to have the exact same participating staff at all different measurement points but that was not possible because all staff were not available at the time of the interviews due to the reasons stated above. The focus groups consisted of a mix of professionals working at the PHC and included physicians, registered nurses, nurse assistants, social workers, psychologists, physiotherapists, and others (social manager, health educator, rehab coordinator, midwife, secretary, behaviourist, head of unit, dietician, secretary, and unknown); the details are presented in Tables 1 and 2. Two of the interviews were conducted as individual interviews because the work situation did not allow more than one person to participate at a time.

### 2.2. Data Collection

A semistructured interview guide [17] was developed by the research team. The guide focused on themes including the staff's experiences with working with risky drinkers and SBI. At the follow-up, themes regarding how their experiences with working with risky drinkers and SBI had changed during the implementation period were added.

All focus group interviews were performed by the same interviewer and were recorded. The interviewer was an occupational therapist with experience in Swedish health care. The interviews took place in a separate room at each of the PHCs where the participants worked except for the two individual interviews, which were performed by phone. Participation was voluntary and the participants were informed that they could abandon participation at any time. The participants were informed about the purpose of the interview and they were encouraged to discuss freely around the themes and to bring their perspectives into the open. The interviews lasted for 23–30 minutes at baseline and 6–19 minutes at follow-up.

### 2.3. Data Analysis

The interviews were recorded and transcribed verbatim. The data were inductively analysed using content analysis, meaning that coding and categorization of data were done in a structured way, gradually deriving the categories from the data in an explorative and descriptive way [17, 18].

The analysis was conducted in several steps with the aim of identifying the experiences of the staff regarding working with risky drinkers and especially how these changed during the implementation process. Initially, the first author listened to all recordings and ensured that the transcripts were accurate. Then all texts were read through several times by the first author to provide a sense of the whole. A qualitative analysis software program, NVivo 10, was used to facilitate the analysis. The first step was performed using open coding by reading the texts line by line to identify meaning units, which were labelled with preliminary codes. The coded meaning units were then combined into preliminary categories based on similarity of content. This first analysis was mainly performed by the first author but continuously discussed with coauthor Fredrik Spak to prevent researcher bias and strengthen the internal validity. Disagreements were discussed until consensus was reached.

In the second analysis step, the purpose and the specific aim of this study were taken into deeper consideration and the analysis process continued with identification of meaning units responding to the aim. The meaning units were then labelled with codes and the codes were compared regarding similarities and differences and then categorized based on similarity of content to build categories.

In the third analysis step, the categories were discussed and then sorted and abstracted into main categories and subcategories that captured the main content of the data with regard to the aim of the study. This deeper analysis of the content was mainly performed by the first author but continuously supervised and discussed with coauthor Ulrika Müssener. The coding and interpretative levels of the categories were continuously discussed with all coauthors to ensure trustworthiness. Quotations were identified to illustrate the categorization and translated from Swedish. In the results, /…/ in a quotation shows that text has been omitted or means a pause or a short silence. [  ] means the authors have added clarification.

## 3. Results

The analysis of the interviews before and after the implementation phases revealed two main categories: (1) awareness of shortcomings, reflecting thoughts before the implementation phases, and (2) change in practice as expressed by the participants after participating in the study (Table 3). Thus, the analysis displayed a pattern of change during the implementation period with regard to awareness, knowledge, and confidence that led to a change in practice. Before the implementation, the participants reported a lack of resources and engagement in working with alcohol and SBI.

### 3.1. Awareness of Shortcomings

Before the implementation period, the participants reported a lack of engagement regarding working with alcohol-related questions. A majority of the participants expressed that they did not work as much with screening and brief interventions as they thought was needed and that they were motivated to do more SBI work but lacked the tools to do so. They also expressed that they lacked knowledge regarding both alcohol and risky drinking and how to advise risky drinkers. This was creating a lack of confidence and insecurity in asking about alcohol, not least because this was regarded as a sensitive issue.

#### 3.1.1. Awareness of Lack of Engagement

Most of the participants seemed to have an awareness of their lack of appropriate engagement despite their positive attitudes.

*I am too bad at asking questions about alcohol actually. I could actually ask questions about alcohol to almost every patient. But I don't do it.*

*I believe health care has an obligation to ask questions about how life styles interfere with health. It should not be strange that health care brings it up; it has to be natural. To not do it is almost misconduct.*

Despite awareness of lack of engagement staff expressed a positive attitude towards implementing SBI. This seemed to be grounded in the belief that the staff actually could facilitate change in patients' alcohol habits. It was also evident that the staff believed that alcohol problems could be the underlying cause of many of the symptoms that patients present at the PHCs and that, through active work on alcohol, they might identify that cause and be able to help patients to a greater extent.

*But it is good in order to identify what is the actual problem with the patient, because it can be alcohol that lies behind a lot of what the patients seek care for.*

There was a wish for alcohol work to be more visible for both patients and staff in order to facilitate the work. The participants thought that if there was advertising material around the PHCs, both staff and patients would remember and embrace the alcohol work to a greater extent. The participants could identify several situations where more systematic alcohol work could be performed and which they regarded as underutilized at the moment. Some participants also expressed motivation to learn more and work more with alcohol prevention and expressed that the implementation efforts that were planned were much needed and perceived that they would be very useful.

*I feel that this is very useful. I want to know more in order to help these people that I encounter.*

#### 3.1.2. Insufficient Knowledge

Lack of knowledge regarding several important issues for alcohol preventive work was expressed among the interviewees. The most highlighted area of lack of knowledge was how to advise risky drinkers. Almost all participants described that they were aware of their shortcomings in responding to risky drinkers and requested more training as planned in the implementation project.

*For some of us it would facilitate with more education in order to understand the limits for risky drinking.*

*It would facilitate us a lot if we knew where to refer patients for additional help when we are insufficient…/…/…And at the same time know what to do by yourself. How far should you go in trying to talk to people before you try to get help elsewhere?*

Lack of knowledge on how to encounter risky drinkers was emphasized and it was revealed that this shortcoming had led to avoidance to ask about alcohol.

*The times I might have avoided asking is because I don't know how to deal with the answer. I don't really know what to do if someone is a risky drinker. How do I deal with that? I simply don't have the knowledge.*

*It can be quite hard if you ask and you get an answer. Can I start a discussion? Do I have enough knowledge to proceed around the alcohol issue? I think that is hard.*

The interviewees believed that the alcohol preventive work would be more efficient if they had more knowledge because patients tend to listen more if they perceive that the person is knowledgeable and confident in discussing the issue.

*I think that the patient understands if you have the right knowledge; they listen more to you. You can explain and talk about it in such a way, with such a tone, that they will take it in a better way and understand.*

#### 3.1.3. Insecurity in Asking

Before the implementation period, most of the participants felt insecure asking about alcohol and intervening with risky drinkers, and this insecurity meant that patients were not being asked about their alcohol habits. One of the factors that contributed to the feelings of insecurity seemed to be the fact that alcohol was regarded as a sensitive issue and that the staff were afraid of offending patients. Some of the interviewees described how they sometimes came up with excuses to not ask about alcohol and that the alcohol questions were easy to forget.

*If you ask the question in the wrong manner, or in a way that the patients experience as offending …//… the relationship can be affected.*

But some staff had a different view and expressed that they were confident in asking about alcohol and had never felt that the patients were offended.

*That it is a sign of us caring. I think it is perceived in that way. I rather think that more patients are dissatisfied because they are not asked.*

Among those who expressed concern about offending patients, some solutions were proposed. They highlighted the importance of patients not feeling singled out and questions about alcohol should be brought up in a natural way or in a natural context. It was suggested that if more patients were asked about alcohol, the issue would become more natural and less sensitive to both the patient and the staff, and the patients would feel less singled out.

*It might be better accepted among the patients if they know that everyone is asked the question, so you they don't feel so singled out and that it is something that we always do.*

### 3.2. Change in Practice

After the implementation period, a majority of the participants expressed that their level of knowledge and their confidence in working with SBI had changed. The participants perceived that they asked more patients about their alcohol habits after the implementation of the project. It was also seen that the PHCs had integrated SBI into routine practice and that the staff asked special patients group more systematically about alcohol. The participants appeared to have been given some of the tools to perform SBI work that they lacked at the baseline. They were also more aware about alcohol both at an individual level and at a PHC level and discussed alcohol more among their colleagues. In general, the participants were positive about continuing to work with alcohol prevention.

#### 3.2.1. Increased Engagement

At the follow-up, many of the participants perceived that the SBI activities at their PHCs had increased. They were convinced that, in general, they asked more patients about alcohol habits after the implementation and had integrated alcohol preventive measures into the daily routines to a greater extent. For example, at some PHCs, the staff have started to systematically ask patients with certain diagnoses; others screened and intervened for alcohol problems systematically at certain visits such as annual health check-ups.

*Yes, of course you ask more frequently, more often, I do that.*

*In fact, I never asked my patients this question before we entered this project, and now it is at every annually check-up….*

Some participants stated that they had started with new tools and approaches, such as health questionnaires or health consultations that included questions about alcohol. Increased systematic work was also evident from more notes about alcohol habits in the medical records.

*You can see a lot more notes in the medical records about the patients' alcohol habits.*

Not only did SBI activities increase during the implementation, but also the participants expressed that they experienced greater general awareness about alcohol and risky drinking at the PHCs and discussed the issue to a greater extent among colleagues at the PHCs after the implementation.

*We talk about alcohol more in general now. Before nobody talked about it.*

#### 3.2.2. Knowledge Gain Changing Practice

The greater awareness of alcohol and establishment of new routines seem to have been mediated by increased knowledge and skills about alcohol prevention. Also, the participants knew more about their own limitations and when and where to refer patients if needed.

*One thing that is important to know is what to do with patients who have a risky dinking behaviour or misuse. I think we have talked a lot about that here at the PHC so it feels like you know what to with the patient.*

They also experienced increased knowledge about alcohol and risky drinking and felt that they knew how to respond to risky drinkers to a greater extent.

*There has been the issue of not knowing what to answer when you get a question back. Now you know a little bit more about where the patient can turn for help and what you can do. Maybe you can't do so much more than say “cut down your intake to half” but you know what advice you can give.*

#### 3.2.3. Confidence in Asking

At the follow-up interviews, it was highlighted by most of the participants that they experienced more confidence in screening and intervening for risky drinking. They expressed that it had become easier to ask patients about their alcohol habits; they dared to ask to a greater extent after the implementation and were more confident about intervening for risky drinking.

*Yes, I believe it feels more secure to ask patients, to bring it up with the patients. Sometimes you can feel that it is sensitive, snooping into their life when it comes to alcohol. But in that way I think it feels a little more secure.*

After the implementation, the participants also expressed that they were more comfortable in persisting with the alcohol issue and that they did not drop the subject if they met resistance from the patient, which implies greater confidence in their own ability to intervene with risky drinkers.

*But I am not as afraid anymore to continue. Before I could stop when I felt, oh, now I am in deep water. I don't anymore, I feel that I can continue, can coax so to speak. Go around and continue. Because if I back off, I confirm for that patient that this is a sensitive issue; if I continue, I can sometimes also explain that we always ask like this when it comes to alcohol because it can lead to pain in the body or bad sleeping habits or….*

The factors that seemed to contribute to this were that they did not regard alcohol as a sensitive issue as they did before and that they had more experience and knowledge as well as more tools.

The fear of offending patients that was expressed at the baseline was not highlighted at all at the follow-up. Many of the participants said that they had never experienced that patients were offended by being asked about alcohol.

One reason that the participants felt contributed to alcohol becoming a less sensitive issue was that the patients felt less singled out when more patients were asked about their alcohol habits.

*And when someone looks a little hesitant or wandering, we say “we ask everyone, both men and women, old and young” so there is nothing odd about that.*

Another factor that seemed to contribute to the decreased insecurity in screening and intervening for risky drinking was increased experience. The participants agreed that the more they worked with these issues, the more confidence they got. They expressed that with more experience it was easier, more natural, and less inconvenient to ask about alcohol.

*It is easier. It gave me experience, practice makes perfect, and if you have done it a couple of times, it becomes more natural to do it. It feels good.*

## 4. Discussion

The aim of this study was to explore how the primary health care staff's experiences of working with risky drinking changed during an implementation process that included two educational sessions and office-based material support. The project focused on inspiring staff to start offering SBI to patients and consequently getting more and more confident in applying SBI. At the baseline, the staff perceived that they did not work enough with SBI although they were motivated to do more. It appears that by participating in the focus group the staff were also given an additional chance to reflect upon their own attitudes and engagement, which might also have contributed to the results. At the follow-up 12 months later, a number of positive changes could be noted. Now, most staff perceived that SBI activities had clearly increased and a number of barriers such as lack of knowledge and confidence in asking about alcohol had been overcome. From the interviews, it became clear that the project had inspired staff to try using the material provided and they had learned by practicing to a great extent.

The lack of knowledge and confidence in bringing up the issue of alcohol was strongly emphasized at the baseline and mentioned as an important reason for not bringing up the issue. The staff also expressed insecurity in how to introduce the issue and how to intervene, as seen previously in most studies [5, 6, 12, 14]. However, this is somewhat surprising since Sweden has made a strong national effort over 5 years to educate large sections of primary health care in SBI from 2004 to 2010 at a total cost of 25 million euros. The project was a government initiative addressing primary, child, maternity, and occupational health care. A multifaceted approach with educational courses, workshops, and seminars was applied in order to encourage learning-by-doing. A number of subprojects involving specific staff categories and settings were undertaken [19]. One important lesson learned from this project was the benefit of involving nurses in SBI in contrast to many other countries. It has been repeatedly suggested that nurses are an underutilized resource in alcohol prevention work [4, 11]. Since the PHCs in this study were representative of PHCs in Sweden, in both rural and urban areas, the lack of knowledge and confidence in working with SBI could probably be generalized to large sections of Swedish primary health care. So the effect of training postgraduate PHC professionals appears to have had limited reach in Sweden despite the great national effort. A better way forward might call for more systematic training in alcohol prevention work in medical schools and nursing schools.

Based on what happened over time in the group of health care professionals participating in the present study, we identified a number of important changes after training and active use of the office-based material during the implementation phases. The health providers in the study felt that both knowledge and confidence had increased at the follow-up and they stated that they had more confidence in bringing up the issue and giving a response and were able to go into the issue in greater depth. This indicates that the implementation was successful in increasing knowledge and confidence and that that effect lasted longer than the implementation period itself. Involving staff other than GPs may explain some of the positive outcomes in the present study.

It was also obvious that some of the staff still regard alcohol as a sensitive issue and that this affected their lack of confidence in talking about alcohol and their reluctance to bring up the issue with patients. The fact that staff consider alcohol a sensitive and difficult issue has been shown in previous studies [20–22]. This is a challenge that must be faced but as some of the caregivers stated, it might be easier for both staff and patients if more patients were asked routinely because it might decrease the risk of causing offense or feelings of being singled out. The fact that the alcohol issue is sensitive was not emphasized as much at the follow-up as at the baseline, indicating that more knowledge, experience, and confidence in talking about alcohol also reduces the sensitiveness of the issue. Staff had started to establish new routines when to ask patients about their alcohol habits and this also decreased the sensitivity of the subject.

Although not measured, most staff stated that more patients were asked about alcohol at the follow-up, indicating success with the frequency of patients being asked, although this was not measured in more exact terms. However, staff expressed decreasing engagement over time; SBI rates increased initially during the implementation phase but they decreased somewhat over time. We studied engagement about 6 months after the last educational sessions and do not know whether the SBI activity will continue to fade out. However, there will probably be a need for repeated booster educational sessions and continuous managerial support. Variability in engagement among staff was noted in the interviews with early adopters and laggers who were more reluctant to adopt a new routine. Some PHC centres decided to systematically screen certain groups of patients and were thus starting to integrate SBI into the daily routines, creating a precondition for continuous engagement over time. Another promising observation that might help establish new routines was increased awareness and discussion among the staff about the negative health consequences of alcohol.

Among the lessons learned from this study, it is obvious that the previous educational efforts extended to the primary care sector in Sweden were not evident. This calls for more effort to integrate alcohol prevention training in medical and nursing schools.

There is some indication that staff were learning by actually doing in practice. Being part of an implementation project with education, new office-based material, and a commitment to try using the new knowledge and tools made them more confident and facilitated a change in practice. The effects of the study seemed to last for at least 6 months after the termination of the study. The positive changes in attitudes and engagement during the study could potentially secure continuous positive development. Also, the decision to specify when to ask patients about their alcohol consumption decreased the sensitivity of asking the question and increased confidence.

In summary, the study shows that staff gain knowledge and confidence in working with alcohol screening and brief intervention when participating in an implementation study with an educational approach. In this study, the effects of participation lasted up to 6 months after the termination of the study. This adds new knowledge to the science of implementation studies concerning alcohol prevention measures, which have otherwise shown disappointing results, emphasizing the importance of practicing and learning in daily meetings with patients/clients [4, 5, 7–9].

### 4.1. Methodological Considerations

In this study, we used an explorative and inductive qualitative approach. A semistructured interview guide was developed and used in order to explore how the perceptions and experiences of working with risky drinkers change over time. We used mainly focus group interviews to gather data because they are an effective method to explore attitudes and needs of professionals and may encourage the participants to share and discuss views, attitudes, and experiences [16–18]. In focus group interviews, there is a risk that participants do not feel free to discuss sensitive or personal experiences and perceptions, especially if they know the other participants, as was the case in this study. However, this could also be seen as an advantage. The focus group interviews facilitated a relaxed discussion and provided a satisfactory framework in that it helped direct the participants toward the issues focusing on them but still allowed the participates to express their answers in their own way.

The focus groups included participants from different categories of staff, which may add to the results with more in-depth perceptions and experiences from professionals with different educational and professional backgrounds sharing their different perspectives [16, 17]. We think it is was a benefit that different professionals were included but one must be aware that it may have affected the results because there is a hierarchy among these professionals [17], even though this was not perceived when listening to and analysing the interviews.

The results predominantly showed that the implementation was successful and staff seem to have been learning-by-doing, gaining more confidence and security in offering SBI. But considering the fact that the PHCs volunteered to participate, as did the staff at the PHCs, this might have led to participants being more positive towards SBI work than occurs in general, thus making successful implementation more likely. Staff who did not participate might have had different views. As always when conducting qualitative research, voluntary participation may lead to the study sample differing from the broader population. One strength in this study was that the interviews revealed both challenges and shortcomings among the participants as well as positive standpoints, indicating that the staff felt they could speak freely. Our data are based on a relatively small sample and a specific group of professionals, and the results cannot be generalized to other groups.

It is a limitation that the authors did not conduct the interviews themselves, that we did not include an observer, and that the moderator of the interviews was not included in the analysis process. This has been addressed by the first author listening to the recorded interviews several times.

Several steps have been taken to ensure the validity of the results. In the present analysis two of the authors (Hanna Reinholdz and Fredrik Spak) read the interview transcripts several times. The meaning units, codes, and categories were discussed by the authors in different combinations and at several points during the process. Interpretation and conclusions were discussed until consensus was reached regarding codes and categories. The authors were of different ages, sexes, and professional backgrounds. The coauthors were all senior researchers and well experienced in either qualitative research or the alcohol research field with focus on early identification and intervention with risky drinkers. Some of the results are supported by previous results indicating an acceptable trustworthiness.

## 5. Conclusions

Staff at PHCs in Sweden are aware of the negative health consequences of alcohol and perceive that they seldom engage in alcohol screening and brief intervention. However, they appear highly motivated to work more actively, which implies that, with the right tools and incentives, a positive change can be achieved as shown in this study. Staff perceived that lack of knowledge and the sensitivity of the topic contribute to low confidence in working with alcohol issues. Participating in an implementation study where staff agree to perform SBI after a training session appears to have continued the learning-by-doing process. Thus, 6 months after the termination of the project, positive attitudes and perceived engagement were prevailing. The more the patients or patient groups who were routinely offered SBI were, the less the staff perceived the alcohol issue to be sensitive, which increased their confidence in bringing up alcohol, leading to a sense of more knowledge, which also facilitated a change in practice.

## Figures and Tables

**Table 1:** Distribution of participants in the focus group interviews at baseline in the SPIRA study.

PHC code	Profession							
	Physician	Registered nurse	Nurse assistant	Social worker	Psychologist	Physiotherapist	Other^a^	Total
A	1	4						5
B	1	3					1	5
C	2	2				1	1	6
D	2	1		1	1		2	7
E	1	1			1			3
F		2	1	1				4
H		2	2					4
J	1	3						4
L		5						5
M		3		1				4
N	1	4						5
R	2	1						3
S	2	2	1				1	6
T	2	3						5
U	2	2		1			1	6
V	2	2			1		1	6

Total	19	40	4	4	3	1	7	78

^a^Other: social manager, health educator, rehab coordinator, unknown, midwife, secretary.

**Table 2:** Participants in the focus group interviews at follow-up in the SPIRA study.

PHC code	Profession							
	Physician	Registered nurse	Nurse assistant	Social worker	Psychologist	Physiotherapist	Other^a^	Total
A	1	1					2	4
B	1	3						4
C		1				2		3
D		2						2
E	1	1			1			3
F		2	1					3
H		2	2					4
J^a^		1						1
L^b^		1						1
M		3		1			1	5
N	1	4					1	6
R	1	1						2
S	3	1	1					5
T	1	2					1	4
U^c^	—	—	—	—	—	—	—	—
V^c^	—	—	—	—	—	—	—	—

Total	9	25	4	1	1	2	5	47

^a^Other: behaviourist, head of unit, dietician, secretary.

^b^Telephone interview.

^c^Did not participate in the follow-up.

**Table 3:** Overview of the main categories and subcategories regarding change in staff's experiences of SBI work during an implementation process.

Main category	Subcategory	Codes
Awareness of shortcomings	Lack of engagement	Attitudes, beliefs, motivation, implementation efforts
	Insufficient knowledge	Shortcomings, encounters, avoidance
	Insecurity in asking	Insecure, excuses, sensitive

Change in practice	Increased engagement	Habits, tools, approaches
	Knowledge gain changing practice	Routines, skills, prevention
	Confidence in asking	Inventing, persisting, increased experiences
